# Coakley on the Nose and Throat

**Published:** 1902-03

**Authors:** 


COAKLEY ON THE NOSE AND THROAT. The Diagnosis and Treatment of Diseases of the Nose, Throat, Naso-Pharynx and Trachea. For the use of Students and Practitioners. By Cornelius G. Coakley, M. D., Professor of Laryngology in the University and Bellevue Hospital Medical College, New York. New (2d) edition. In one handsome 12mo. volume of 556 pages, with 103 engravings and 4 colored plates. Cloth, $2.75 net. Lea Brothers & Co., Philadelphia and New York. 1901.

The first edition of this work appeared in 1899, and became widely used. The second edition now comes forth, and will be appreciated by the profession. A careful revision, and some additions, have been made. A new chapter on the affections of the upper respiratory tract in the infectious diseases has been added.

The work is especially adapted to the general practitioner. It has been said of this book that there is not a paragraph of nonessential matter in the whole volume. T. J. B.

